# Differentiation of physiologic versus pathologic basal septal fibrosis: Proposed diagnostic criteria and associations with clinical and CMR-based markers of cardiovascular disease

**DOI:** 10.1186/1532-429X-16-S1-P104

**Published:** 2014-01-16

**Authors:** Sebastien X Joncas, Louis Kolman, Carmen Lydell, Sarah Weeks, Andrew G Howarth, Naeem Merchant, James A White

**Affiliations:** 1 Stephenson Cardiovascular Magnetic Resonance Centre, University of Calgary, Calgary, Alberta, Canada; 2 Université de Sherbrooke, Quebec, Quebec, Canada

## Background

**Figure 1:**
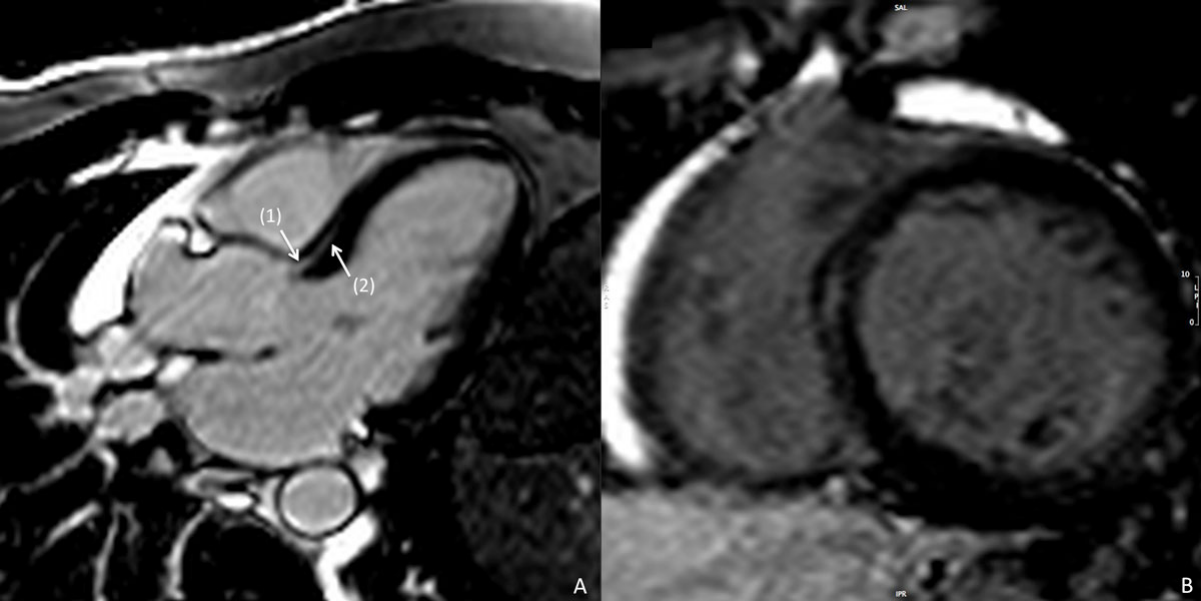
**(A) Apical long axis 3-chamber late gadolinium enhancement (LGE) image in a patient with "physiologic basal septal LGE" according to the criteria of; (1) direct contact with the aortic root, and (2) decreasing signal intensity apically**. (B) Basal short axis view with mid myocardial late gadolinium enhancement.

Abnormal late gadolinium enhancement (LGE) is commonly identified in the basal septum of patients with dilated cardiomyopathy, hypertrophic cardiomyopathy and sarcoidosis, and has been associated with major adverse events. However, basal septal LGE may also be seen in otherwise normal individuals (Figure 1) and may be "physiologic". No diagnostic criteria to differentiate the latter have been established, and both its prevalence and clinical significance remain uncertain. In this study we propose such criteria and examine its prevalence and association with markers of cardiovascular disease.

## Methods

A total of 615 consecutive LGE CMR studies were evaluated. Patients with prior valvular surgery (n = 91) or congenital heart disease (n = 94) were excluded, resulting in 430 studies. All were blindly scored for the presence of "physiologic septal LGE" according to pre-defined criteria, as follows; (1) LGE in direct contact with the aortic root, AND (2) decreasing in signal intensity towards the apex (see Figure 1). Baseline clinical and CMR-based measures of structural heart disease, inclusive of total LGE volume (> 5SD threshold) were compared between those with and without diagnostic criteria being met.

## Results

Mean age and LVEF of the entire population were 55.8 ± 14.5 years and 49.1 ± 21.7%, respectively. A total of 73 patients (20.4%) met criteria for "physiologic basal septal LGE". As shown in Table 1 no association of this finding with any other clinical or CMR markers of cardiovascular disease was identified. The only identified difference identified among those with the finding was a slightly higher LV ejection fraction (53.8 ± 19.4 vs. 48.1 ± 22.2, p = 0.045) and age at time of imaging (59.2 ± 12.7 vs. 55.1 ± 14.9, p = 0.030). No association was seen between the presence of "physiologic basal septal LGE" and total LGE (inclusive of all patterns of disease), measuring 10.9 ± 14.3% by signal-threshold based quantification. By comparison, those patients having basal septal LGE but not meeting "physiologic" criteria showed a significantly lower mean LVEF of (30.2 ± 14.6% (p < 0.001) and higher total burden of LGE (13.4 ± 14.9%, p = 0.025). A similar prevalence of "physiologic basal septal LGE" was found in a population of 35 healthy volunteers.

**Table 1:** Clinical and CMR characteristics of patients with and without "physiologic basal septal LGE".

	"Physiologic basal basal LGE"		p-value
**Clinical variables**	**Present (n = 73)**	**Absent (n = 357)**	

Age (years)	59.2 ± 12.7	55.1 ± 14.9	0.030

Male (n, %)	51 (69.9)	230 (64.4)	0.374

BMI (g/m2)	29.0 ± 5.1	28.2 ± 5.5	0.215

GFR (mL/min)	82.3 ± 22.1	77.4 ± 22.1	0.098

Smoking (n, %)	19 (26.0)	105 (29.4)	0.561

Hyperlipedemia (n, %)	29 (39.7)	140 (39.2)	0.935

Diabetes (n, %)	10 (13.7)	61 (17.1)	0.477

Hypertension (n, %)	32 (43.8)	139 (38.9)	0.436

History of coronary disease (n, %)	20 (27.4)	129 (36.1)	0.153

NYHA class			

Class I - II (n, %)	23 (63.9)	140 (61.9)	

Class III - IV (n, %)	13 (36.1)	86 (38.1)	0.823

**CMR variables**			

- LV anteroseptal wall thickness (mm)	10.8 ± 3.5	10.8 ± 4.1	0.963

- LV end diastolic volume index (mL/m2)	86.2 ± 40.4	97.0 ± 43.2	0.051

- LV mass index (mL/m2)	81.3 ± 27.6	86.2 ± 28.8	0.185

- LV ejection fraction (%)	53.8 ± 19.4	48.1 ± 22.2	0.045

- RV end diastolic volume index (mL/m2)	63.2 ± 18.2	65.8 ± 24.5	0.374

- RV ejection fraction (%)	57.7 ± 13.0	54.0 ± 15.5	0.061

- Presence of LGE (n, %)	41 (56.2)	243 (68.1)	0.050

- Total fibrosis (≥ 5 SD threshold) (mL)	10.6 ± 14.9	12.4 ± 17.9	0.440

- Percentage of fibrosis (≥ 5 SD threshold) (%)	9.8 ± 12.9	11.1 ± 14.5	0.44

Data are means ± standard deviation. BMI = body mass index. LV = left ventricle. RV = right ventricle. LGE = Late gadolinium enhancement.

## Conclusions

Among a large CMR referral population "physiologic basal septal LGE" was identified in 20% of all patients. This finding does not show association with any clinical or CMR-based marker of cardiovascular disease. While outcome-based studies are both preferred and required, the proposed criteria for "physiologic basal septal LGE" provides a practical tool for the differentiation of benign versus pathologic septal LGE in patients referred for CMR.

## Funding

None.

